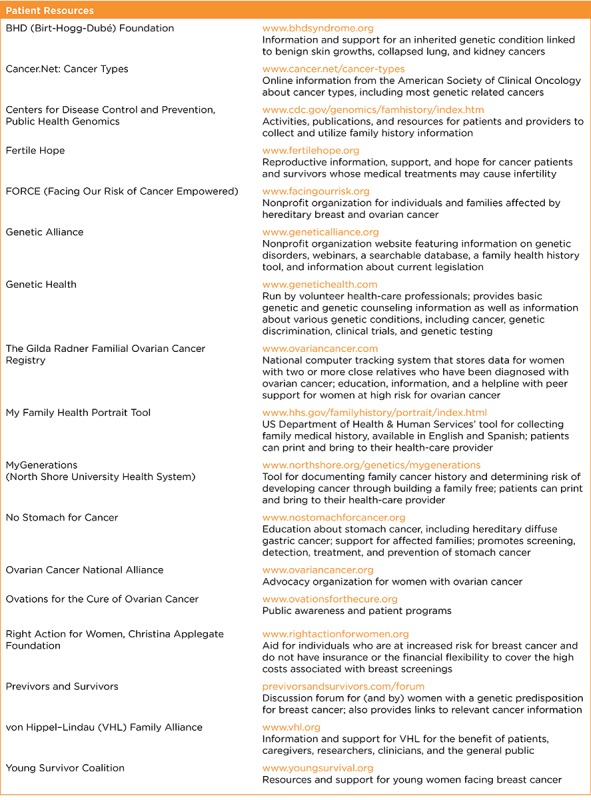# Cancer Genetic Resources

**Published:** 2013-09-01

**Authors:** Wendy H. Vogel, Deborah F. Pencarinha

**Affiliations:** Ms. Vogel is a nurse practitioner and Ms. Pencarinha is a genetic counselor, both at Wellmont Cancer Institute in Kingsport, Tennessee.

Human genetics is a complicated science. There are a multitude of terms to understand, such as cancer genetics, hereditary genetics, cellular genetics, genetic engineering, human genome, predictive genetic testing, Mendelian inheritance, and many more. If we, as advanced practitioners (APs) in oncology, are sometimes confused, imagine what our patients feel.

## Cancer and Genetics

All cancer is genetic, but not all cancer is hereditary. In oncology, APs deal primarily with two types of genetic testing: tumor genetics and hereditary genetics. Tumor genetics is the study of acquired mutations of certain cells in the body. These mutations may cause abnormal cell growth and division, thus creating a tumor that continues to grow and invade other body tissues. These mutations cannot be passed to offspring, as they are only present in the cancerous cells. Some cancer treatments will target the cause of the harmful mutation. An example of this would be current treatments for chronic myelogenous leukemia, such as imatinib, which targets the bcr-abl oncogene: the result of an acquired mutation.

Hereditary genetics is the study of mutations that are present in every cell of the body and thus may be passed on to offspring. Each of us has two copies of every gene: one from our mother and one from our father. We could inherit a defective gene from our parent and in turn pass it on to a child. For example, the BRCA1 gene functions as a cancer suppressor, protecting both men and women from breast cancer (and in women, from ovarian cancer as well). When a defective BRCA1 gene is present, we have less protection. If the other BRCA1 gene (inherited from our other parent) breaks (mutates), we no longer have protection against breast or ovarian cancer. Not everyone with a BRCA1 mutation develops cancer, but the risk is significantly increased (up to 87% lifetime risk of breast cancer).

## Resources

As APs, we must have a basic knowledge of genetics. We help our patients understand the difference between the types of genetics and how that affects their disease and their treatment. We need to identify those patients and family members who would benefit from genetic counseling and testing. The partnership between the AP and the genetic counselor synergizes cancer genetics expertise with medical and clinical knowledge to further the best outcomes for the patient. In this article, you will find a collection of valuable Internet resources intended for us, as APs, as well as for our patients. The resources intended for patients appear grouped together in this table. Please feel free to reproduce the list for your patients or download a copy from the JADPRO website.

**Table 1:**
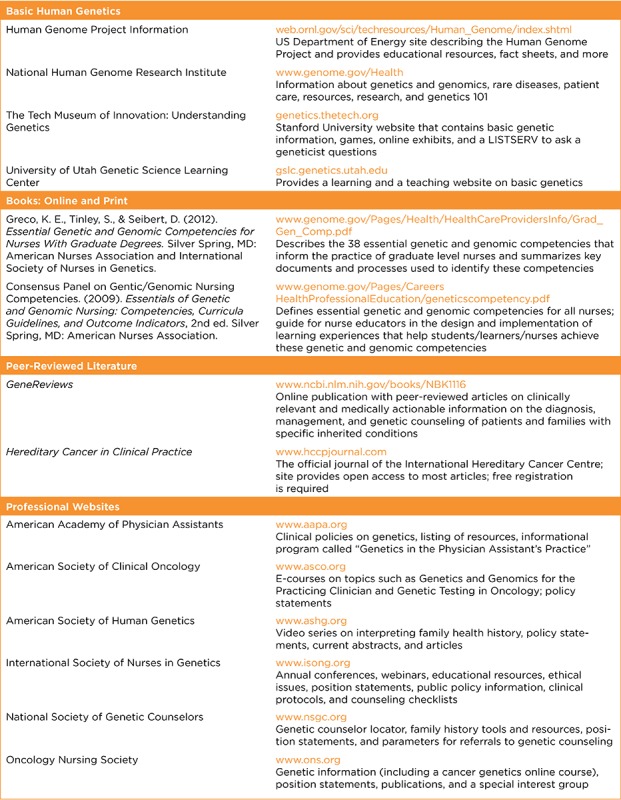


**Table 1 (contd.):**
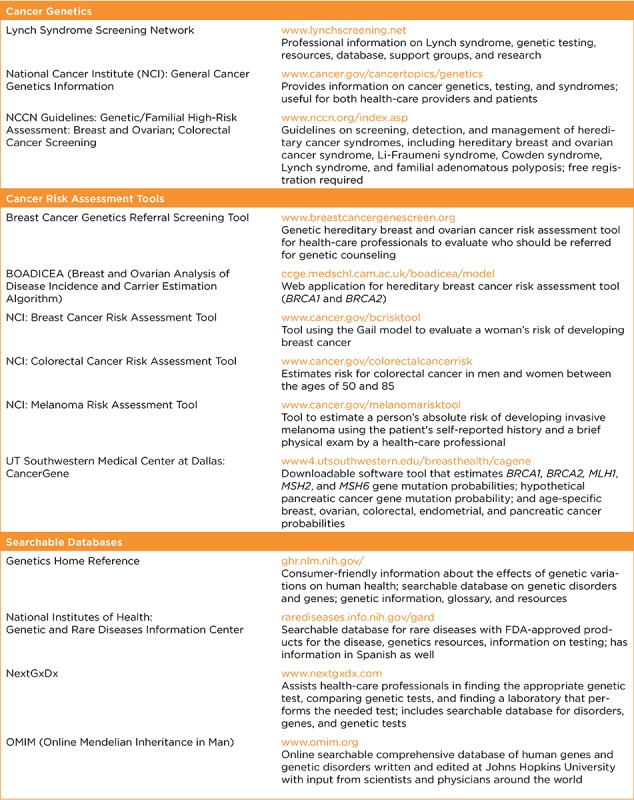


**Table 1 (contd.):**